# Regulation of Airway Inflammation by G-protein Regulatory Motif Peptides of AGS3 protein

**DOI:** 10.1038/srep27054

**Published:** 2016-06-07

**Authors:** IL-Whan Choi, Do Whan Ahn, Jang-Kyu Choi, Hee-Jae Cha, Mee Sun Ock, EunAe You, SangMyung Rhee, Kwang Chul Kim, Yung Hyun Choi, Kyoung Seob Song

**Affiliations:** 1 Department of Microbiology, Inje University College of Medicine, Busan, Korea; 2 Department of Physiology, Kosin University College of Medicine, Busan, Korea; 3 Department of Parasitology and Genetics, Kosin University College of Medicine, Busan, Korea; 4 Institute of Medicine, Kosin University College of Medicine, Busan, Korea; 5 Department of Life Science, Chung-Ang University, Seoul, Korea; 6 Department of Otolaryngology-Head and Neck Surgery, University of Arizona College of Medicine, Tucson, AZ, USA; 7 Department of Biochemistry, College of Korean Medicine, Don-Eui University, Busan, Korea

## Abstract

Respiratory diseases such as asthma, chronic obstructive pulmonary disease (COPD), and lung infections have critical consequences on mortality and morbidity in humans. The aims of the present study were to examine the mechanisms by which CXCL12 affects *MUC1* transcription and airway inflammation, which depend on activator of G-protein signaling (AGS) 3 and to identify specific molecules that suppress CXCL12-induced airway inflammation by acting on G-protein-coupled receptors. Herein, AGS3 suppresses CXCL12-mediated upregulation of *MUC1* and *TNFα* by regulating Gα_i_. We found that the G-protein regulatory (GPR) motif peptide in AGS3 binds to Gα_i_ and downregulates MUC1 expression; in contrast, this motif upregulates TNFα expression. Mutated GPR Q34A peptide increased the expression of MUC1 and TGFβ but decreased the expression of TNFα and IL-6. Moreover, CXCR4-induced dendritic extensions in 2D and 3D matrix cultures were inhibited by the GPR Q34A peptide compared with a wild-type GPR peptide. The GPR Q34A peptide also inhibited CXCL12-induced morphological changes and inflammatory cell infiltration in the mouse lung, and production of inflammatory cytokines in bronchoalveolar lavage (BAL) fluid and the lungs. Our data indicate that the GPR motif of AGS3 is critical for regulating MUC1/Muc1 expression and cytokine production in the inflammatory microenvironment.

Although the importance of mucin proteins has been established, the physiological functions of mucins are still unclear. Recent progress in airway mucin research has revealed that the role of mucins during airway infection is not limited to mucociliary clearance^1^. Understanding the characteristics of mucins that control the production of inflammatory cytokines and subsequent down-regulation of airway inflammation in respiratory diseases is essential for developing novel therapeutic compounds against inflammation. Under disease circumstances, uncontrolled mucin hyperproduction and hypersecretion can modulate cytokine production to increase or decrease airway inflammation. Thus, elucidating of the intracellular mechanisms underlying the negative regulation of cytokine-induced mucin overproduction should reveal important clues toward understanding airway mucous hypersecretion^2^,^3^.

MUC1 is a transmembrane mucin glycoprotein that is expressed on the apical surfaces of mucosal epithelial cells^4^. Kim *et al.* reported that MUC1 attenuates epithelial inflammation in response to a common mucosal pathogen, and that Muc1 deficiency leads to lung injury during chronic bacterial infection in mice^5^,^6^,^7^,^8^. Thus, MUC1 may have an essential role in the attenuation of respiratory inflammation, and MUC1 dysfunction likely contributes to the pathogenesis of chronic inflammatory respiratory disease^8^. However, the precise relationship between chemokines and MUC1 during airway inflammation and the mechanisms by which chemokines, their receptors, and MUC1 interact have not yet been identified.

Coupling of stromal cell derived factor 1 (SDF-1a; CXCL12) with its receptor, chemokine (C-X-C motif) receptor (CXCR) 4, plays a critical role in inflammation, as well as in cancer metastasis^9^. Although several reports have indicated that CXCR4/CXCL12 signaling induces inflammation, Fan *et al.* reported that CXCR4/CXCL12 signaling actually activates anti-inflammatory signaling pathways and suppresses inflammation^10^. However, no studies have yet described the relationship between CXCR4 and MUC1/Muc1.

Activators of G-protein signaling (AGS) proteins are involved in a wide spectrum of biological activities, enabling tissues to respond and adapt to various physiological and pathological challenges^11^. Of all the AGS proteins, the functions of AGS1 and AGS3 are the most well established^12^. These AGS proteins bind to Gα_i_-GDP; moreover, activation of Gα_i_-GDP is independent of GPCR signaling. AGS proteins were identified in a functional screen for cDNA sequences whose products activated G-protein signaling in the absence of a seven-membrane span receptor (receptor-independent activator of G-protein signaling)^13^,^14^. AGS3 has been shown to bind to and stabilize inactived Gα_i_, which augments signaling through Gβγ-mediated effectors; however, this interaction also inhibits signaling through Gα_i_-coupled receptors and prohibits G-protein heterotrimer reformation and receptor reassociation^15^. AGS3 possesses seven N-terminal tetratricopeptide repeats (TPRs), which are followed by four conserved C-terminal G-protein regulatory motifs, known as GPR or GoLoco motifs^16^,^17^. The four C-terminal GPR motifs of AGS3 are both necessary and sufficient for G-protein dissociation inhibitor (GDI) activity on Gαi by binding GDP-bound Gα_i_^14^. AGS3 has been shown to be highly expressed in the brain and testes^18^. However, no reports have yet described whether AGS3 is expressed in the lung, and the effects of AGS3 on airway inflammation have not yet been determined.

In the present study, we examined the role of chemokine/GPCR-mediated induction of MUC1 expression on airway inflammation and identified a small molecule that upregulates MUC1 expression and decreases airway inflammation.

## Results

### CXCL12 induces MUC1 expression via the Gα_i_-coupled CXCR4 receptor

**Figure 1:**
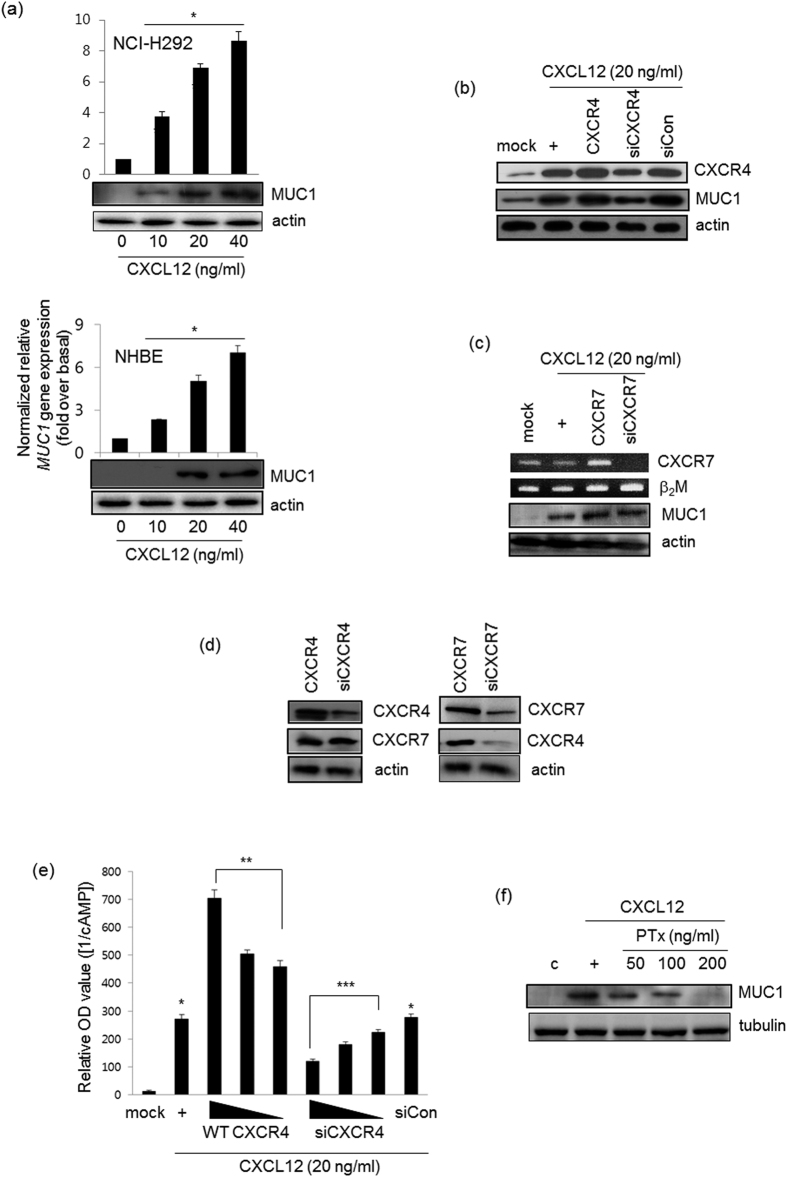
CXCL12 induces MUC1 expression via the CXCR4 receptor in NCI-H292 cells. (**a**) Confluent and quiescent NCI-H292 cells (upper panel) and NHBE cells (lower panel) were treated for 24 hours with various concentrations of CXCL12, and then lysates were harvested and analyzed by real-time quantitative RT-PCR and Western blot for MUC1 transcript and protein, respectively. *p < 0.05 compared to the control. After cells were transfected with either construct expressing wild-type CXCR4 or the siRNA constructs of CXCR4 (**b**,**c** for CXCR7), cells were treated with CXCL12 for 24 hours prior to the collection of total RNA for conventional RT-PCR (for CXCR4 transcript) and real-time quantitative RT-PCR. *p < 0.05 compared to the control, **p < 0.05 compared to CXCL12 treatment only, and ***p < 0.05 compared to CXCR4 transfection. β_2_M, Beta-2- microglobulin, was used as a loading control. (**d**) After transfection with either wild-type CXCR4 or siRNA-CXCR4, cells were harvested and performed Western blot analysis for CXCR7 transcript (left panel). And either wild-type CXCR7 or siRNA-CXCR7 construct was transfected to Western blot for CXCR4 (right panel). (**e**) The cells were transiently transfected with the construct expressing wild-type CXCR4 (0.1, 0.5, or 1.0 μg) or siRNA-CXCR4 (5, 25, or 50 nM). Cells were serum-starved overnight and then treated with the indicated concentrations of CXCL12 for four hours, after which cAMP production was measured. The values shown are means ± SD of experiments performed in triplicate. *p < 0.05 compared to the control, **p < 0.05 compared to CXCL12 treatment only, and ***p < 0.05 compared to CXCR4 transfection. (**f**) Cells were treated with PTx with different dosages for 4 hr, and then stimulated for 24 hr with CXCL12 prior to collection of total lysates for Western blot analysis for MUC1 expression with anti-MUC1 against C-terminal region. These figures are representative of three independent experiments. All of uncropped gels (full-length gels) are located in supplemental figures.

We hypothesized that CXCL12 might induce *MUC1* gene expression to alter inflammatory microenvironment in the airway. CXCL12 increased MUC1 mRNA expression and protein production in a dose-dependent manner in both human airway epithelial cells (NCI-H292 cells) and normal human bronchial epithelial (NHBE) cells (Fig. 1a). In addition, MUC1 mRNA and protein levels reached their peak at 12 hrs; these levels were maintained for 24 hours (data not shown). We examined whether CXCL12 mediates coupling of CXCR4 to Gα_i_. Of the CXCR receptors, CXCR4 and CXCR7 are expressed well in airway epithelial cells^19^,^20^. Whereas wild-type CXCR4 increased CXCL12-induced *MUC1* gene expression, siRNA-CXCR4 dramatically diminished *MUC1* gene expression (Fig. 1b). Interestingly, CXCR4 was essential for CXCL12-induced *MUC1* gene expression, while CXCR7 was not critical for it (Fig. 1c). Next, we investigated whether overexpression of CXCR4 or CXCR7 affects CXCR7 or CXCR4 expression, respectively. siRNA-mediated silencing of CXCR4 had no effect on CXCR7 expression and vice versa (Fig. 1d). We next measured adenyl cyclase activity after CXCL12-treated cells were transfected with either wild-type CXCR4 or siRNA-CXCR4. As expected, overexpression of wild-type CXCR4 dramatically inhibited cyclic AMP production in a dose-dependent manner, whereas siRNA-CXCR4 increased cAMP production (Fig. 1e). For the same reason, we sought to investigate whether Gα_i_ signaling may be essential for *MUC1* gene expression, Pertussis toxin (PTx), Gα_i_ inhibitor, was utilized. *MUC1* gene expression was inhibited by PTx in a dose-dependent manner (Fig. 1f). Taken together, our results suggest that CXCL12 induces MUC1 expression via CXCR4/Gα_i_ signaling in airway epithelial cells.

### AGS3 downregulates MUC1 expression and decreases airway inflammation

**Figure 2:**
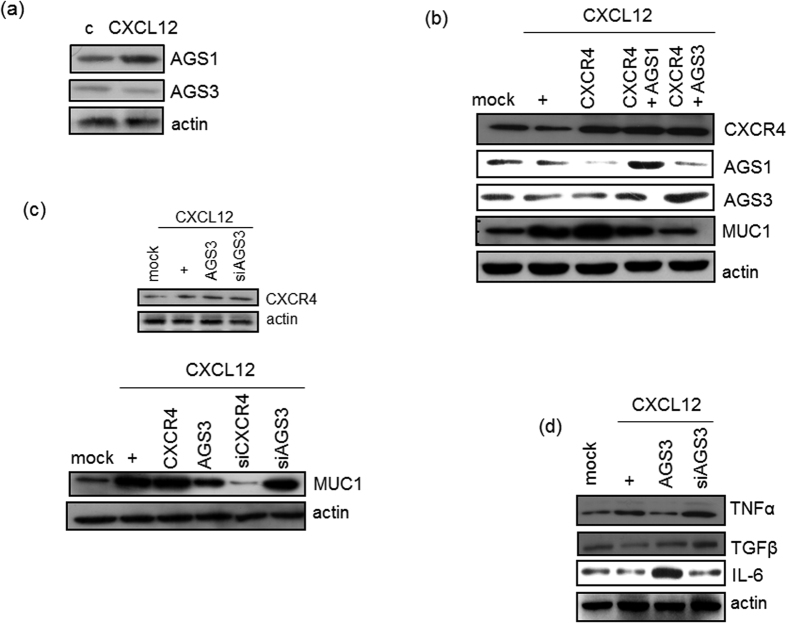
Effect of AGS3 on CXCL12-induced airway inflammation. (**a**) Confluent and quiescent NCI-H292 cells were treated for 24 hours with CXCL12 (20 ng/ml). Cell lysates were then prepared, and the levels were analyzed by RT-PCR. (**b**) Cells were transfected with CXCR4 and either AGS1 or AGS3. Cells were then treated with CXCL12 for 24 hours prior to the isolation of total RNA for conventional RT-PCR (for CXCR4, AGS1, and AGS3) and real-time quantitative RT-PCR (for MUC1). *p < 0.05 compared with the control; **p < 0.05 compared with the CXCL12 treatment alone; ***p < 0.05 compared with CXCR4-transfected cells. Cells were transfected with a construct driving either the expression of wild-type CXCR4 or AGS3 or with siRNA specific for either CXCR4 or AGS3. Cells were then treated with CXCL12 for 24 hours (**c**) or four hours (**d**) prior to the isolation of total RNA for conventional RT-PCR and real-time quantitative RT-PCR. *p < 0.05 compared with the control; **p < 0.05 compared with CXCL12 treatment alone; ***p < 0.05 compared with CXCR4-transfected cells. Data shown are representative of three independent experiments.

We assessed whether AGS1 and AGS3 regulate airway inflammation. First, we investigated whether CXCL12 induces *MUC1* expression, with the hypothesis that this might enable the recruitment of inflammatory cytokines/mediators. We next investigated whether CXCL12 induces *AGS1* and *AGS3* expression in NCI-H292 cells using RT-PCR analysis. Unexpectedly, the expression of *AGS1* and *AGS3* was not affected by CXCL12 stimulation (Fig. 2a). We next determined whether ectopic expression of AGS affects CXCL12/CXCR4-induced *MUC1* expression by cotransfecting cells with wild-type CXCR4 and either AGS1 or AGS3. AGS3 overexpression dramatically inhibited CXCL12/CXCR4-mediated induction of *MUC1* expression compared to AGS1 overexpression, indicating that AGS3 appears to have more functional activity than the AGS1, at least in NCI-H292 cells (Fig. 2b). Interestingly, either AGS1 or AGS3 did not affect CXCL12-induced *CXCR4* gene expression (Fig. 2b, upper panel). AGS1 has not any GPR motif, but AGS3 has four GPR motifs^12^. As AGS3 inhibited much more *MUC1* gene expression than AGS1, we selected AGS3 as suppressor protein for further studies. To examine whether the overexpression and siRNA of AGS3 can affect CXCR4 production and *MUC1* gene expression, CXCL12 was treated for 24 hours (Fig. 2c). The AGS3 overexpression and knock-down did not affect CXCR4 production (Fig. 2c, upper panel). In addition, whereas overexpression of wild-type AGS3 inhibited CXCL12-mediated induction of *MUC1* expression, this induction was restored with AGS3-specific siRNA. Moreover, overexpression of AGS3 downregulated *TNFα* expression; in contrast, siRNA-mediated silencing of AGS3 upregulated *TNFα* expression. In contrast, opposite effects were observed with the anti-inflammatory cytokine, *TGFβ* (Fig. 2d). Of particular note, overexpression of AGS3 upregulated *IL-6* expression, whereas siRNA-mediated silencing of AGS3 downregulated *IL-6* expression. These results suggest that AGS3 functions as a partial suppressor that is able to downregulate the expression of certain inflammatory cytokines in the airway, but not *IL-6*.

### The CXCL12-activated CXCR4 receptor binds to MUC1 and upregulates IL-6 expression

**Figure 3:**
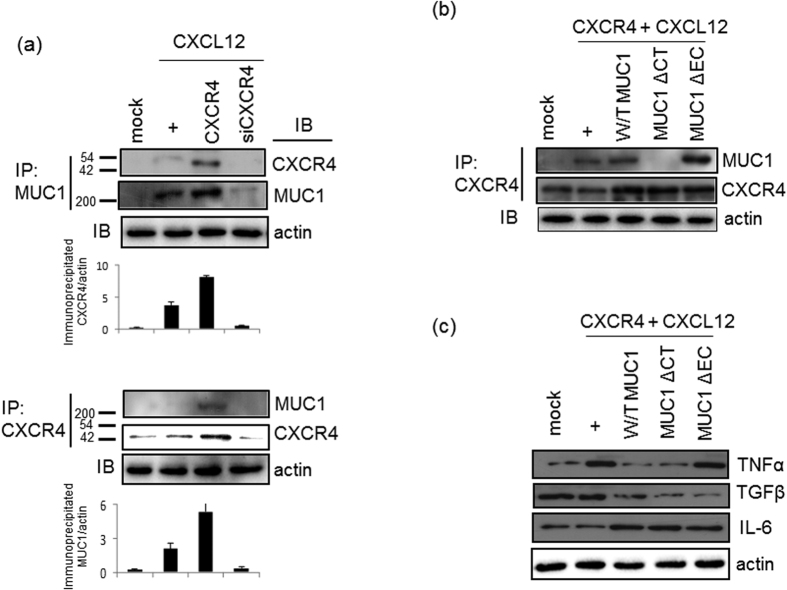
The interaction between CXCR4 and MUC1 downregulates *TNFβ* expression, but not *IL-6* expression. (**a**) NCI-H292 Cells were treated for four hours, and then cell lysates were prepared. Immunoprecipitations were performed with anti-total-MUC1 or anti-CXCR4 antibody, and the eluted proteins were immunoblotted with anti-CXCR4 or anti-total-MUC1 antibody. IP, immunoprecipitation; IB, immunoblotting. (**b**) Confluent and quiescent cells were cotransfected with a construct driving the expression of CXCR4 and a construct driving the expression of wild-type MUC1, MUC1ΔCT, or MUC1ΔEC. Cells were then treated for four hours with CXCL12. CXCR4 was then immunoprecipitated from total cell lysates with anti-CXCR4 antibody, and the eluted proteins were immunoblotted with anti-total-MUC1 antibody. The membrane was then stripped and reprobed with anti-CXCR4 antibody (**c**) At 48 hours post-transfection, cells were stimulated with CXCL12 for four hours. Cell lysates were then generated prior to RT-PCR analysis with specific primers. Data shown are representative of more than three independent experiments.

To investigate the role of MUC1 in CXCL12-activated CXCR4 signaling, immunoprecipitations were performed with anti-MUC1 antibodies (Fig. 3a). In NCI-H292 cells transfected with wild-type CXCR4, the binding of CXCL12 to CXCR4 increased the amount of immunoprecipitated MUC1; however, this interaction did not occur in cells transfected with CXCR4-specific siRNA. Similarly, anti-CXCR4 antibodies efficiently immunoprecipitated CXCR4 from cell lysates and also coimmunoprecipitated MUC1. MUC1 is an anti-inflammatory mucin; accordingly, expression of the major inflammatory cytokine, TNFα, has been shown to be downregulated by MUC1 overexpression^5^,^6^,^21^. Thus, we hypothesized that the increased MUC1 expression resulting from the activation of CXCR4 might decrease the expression of inflammatory cytokines in our system. To examine this hypothesis, we generated a cytoplasmic tail deletion mutant (MUC1ΔCT) and an extracellular domain deletion mutant (MUC1ΔEC)^22^,^23^. Whereas MUC1ΔEC efficiently bound to CXCR4, MUC1ΔCT did not, indicating that the cytoplasmic tail of MUC1 mediates its binding to CXCR4 (Fig. 3b). To determine whether the cytoplasmic tail is essential for the physiological function of MUC1, we transfected with either wild-type MUC1 or two different mutant MUC1 constructs into cells, respectively (Fig. 3c). Compared with cells transfected with either wild-type MUC1 or MUC1ΔCT, cells transfected with MUC1ΔEC exhibited increased *TNFα* expression after the treatment of CXCL12 for 4 hours. This finding indicates that the cytoplasmic tail of MUC1 plays a physiological role in the regulation of airway inflammation. Interestingly, expression of the anti-inflammatory cytokine TGFβ was decreased in cells transfected with either MUC1ΔCT or MUC1ΔEC compared with cells transfected with wild-type MUC1. Additionally, the expression of another inflammatory cytokine, IL-6, was increased in the cells transfected with either wild-type MUC1 or the MUC1ΔCT deletion mutant. In contrast, the expression of *IL-6* was slightly decreased upon transfection with MUC1ΔEC. Moreover, CXCR4 stimulation alone did not upregulate *IL-6* expression. Cumulatively, these results suggest that the extracellular domain of MUC1 is critical for MUC1-mediated downregulation of *TNFα* expression; in contrast, the extracellular domain of MUC1 increased *IL-6* gene expression in the airway.

### The mutant GPR motif of AGS3 is a novel suppressor peptide in airway epithelial cells

**Figure 4:**
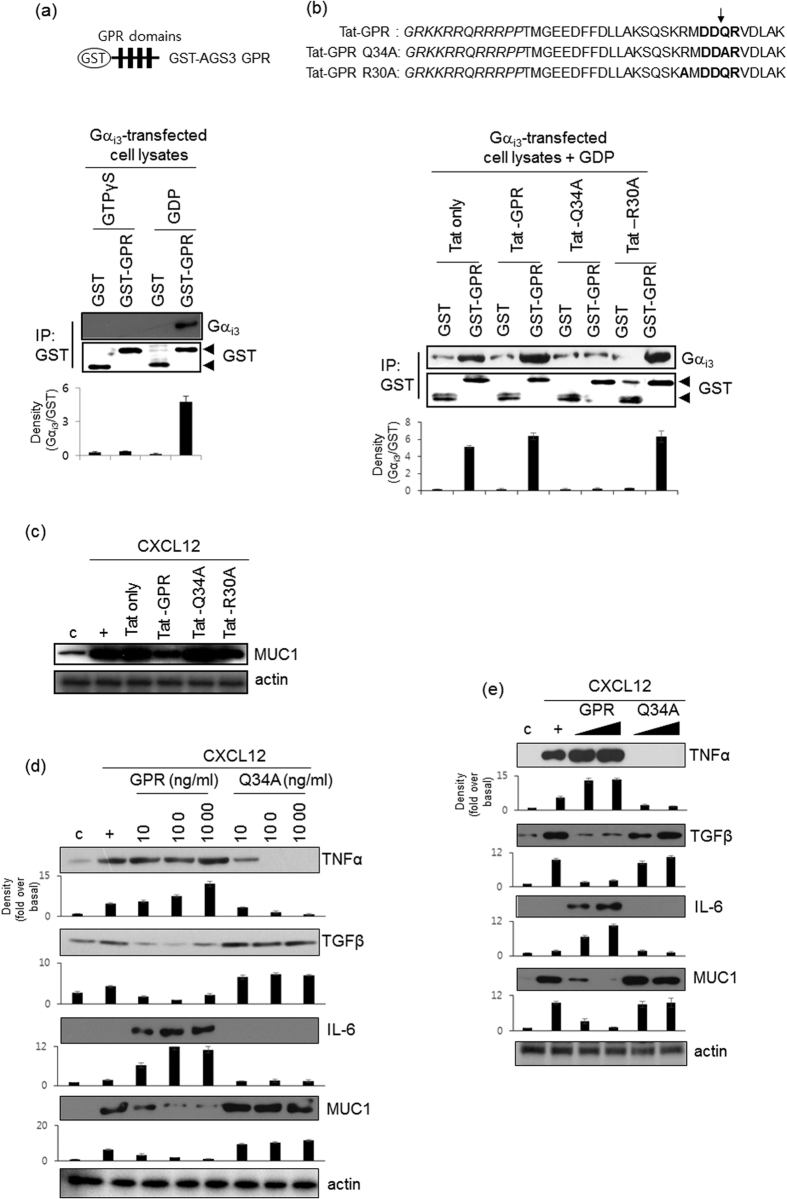
Effect of GPR peptides on CXCL12-mediated induction of gene expression. (**a**) *In vitro* GST pull-down assay using recombinantly produced GST-AGS3-GPR proteins as exogenous binding partners. Cell lysates were generated from NCI-H292 cells transfected with a construct driving the expression of Gα_i3_ and lysates were then loaded with 100 μM GTPγS or 100 μM GDP and 25 mM MgCl_2_ at 24 °C for 30 min. The lysates were then incubated with 250 nM GST fusion proteins for 30 min, and the bound proteins were analyzed by immunoblotting with specific Gα_i3_ and GST antibodies. (**b**) Tat-only, Tat-GPR, Tat GPR Q34A, and Tat GPR R30A peptides (100 ng/ml, each) was added into the assay mixture (100 μM GDP and 25 mM MgCl_2_) at 24 °C for 30 min. The lysates were then incubated with 250 nM GST fusion proteins for 30 min, and the bound proteins were analyzed by immunoblotting with specific antibodies. (**c**) Cells were treated with Tat, Tat-GPR, Tat-Q34A, or Tat-R30A peptide for 24 hours and then treated with CXCL12 for either 24 hours. We performed real-time quantitative RT-PCR. *p < 0.05 compared with the control; **p < 0.05 compared with Tat-only peptide treatment alone; ***p < 0.05 compared with Tat-GPR peptide treatment. Data shown are representative of three independent experiments. (**d**) Cells were treated with either the consensus GPR peptide or the Q34A peptide for 24 hours and then treated with CXCL12 for either 4 hours (cytokine expression) or 24 hours (MUC1 expression). Cell lysates were then prepared prior to Western blot analysis. The Y-axis in graph is expressed as the fold over basal and is presented as the mean ± SD of three experiments. (**e**) Confluent and quiescent NHBE cells were treated with either the consensus GPR peptide or the Q34A peptide (0.1 and 1.0 μg/ml, respectively) prior to treatment with CXCL12 (20 ng/ml) for either 4 hours (cytokine expression) or 24 hours (MUC1 expression). Cells were then harvested, and total lysates were prepared prior to Western blot analysis. Data shown are representative of three independent experiments.

AGS3 contains seven tetratricopeptide repeats (TPRs) and three G-protein regulatory (GPR) motifs. To investigate whether the GPR motif binds to GDP-bound Gα_i_ in NCI-H292 cell lysates, we generated a recombinant GST-GPR fusion protein harboring four GPR motifs. In lysates from cells transfected with a Gα_i3_ overexpression construct, GST-GPR efficiently captured GDP-bound Gα_i_ (Fig. 4a). We determined whether GPR motif has critical physiological functions to regulate inflammatory cytokines expression and binds to Gα_i_. Thus, we generated a GPR peptide consisting of 28 amino acids and a TAT (GRKKRRQRRRPP) sequence to enable cell permeability. In addition, we generated a mutant peptide (Tat-GPR Q34A) in which the core sequence of the GPR motif (DDQR) was changed to DDAR (Fig. 4b)^24^,^25^,^26^. Tat-GPR induced the binding of GST-GPR to Gα_i3_ protein and, conversely, Tat-Q34A peptides blocked the binding of GST-GPR to Gα_i3_ protein due that Tat-Q34A peptide could not bind to Gα_i3_ protein. To investigate whether the effect of GPR or Q34A peptides might be non-specific binding or has an artificial effect, we synthesized GPR-R30A peptides (non-GPR motif core sequence; hereinafter R30A). R30A peptides and GPR peptides had the same binding effect. We also checked *MUC1* gene expression using several peptides (Fig. 4c). GPR and R30A peptides dramatically decreased *MUC1* gene expression; however, Q34A peptide increased *MUC1* gene expression, suggesting that the GPR core sequence may play a critical role for its own function. In addition, in order to determine whether the GPR peptide modulates the expression of MUC1 and inflammatory cytokines in the airway, cells were exposed to either Tat-GPR or Tat-GPR Q34A peptide for four hours prior to treatment with CXCL12. Exposure to Tat-GPR peptide increased the expression of *TNFα* and *IL-6*; however, the expression of these two cytokines was decreased by Tat-GPR Q34A peptide in a dose-dependent manner until baseline expression was reached (Fig. 4d). Interestingly, GPR Q34A peptide also upregulated *TGFβ* expression. Moreover, the consensus GPR peptide downregulated *MUC1* expression, whereas Q34A peptide upregulated *MUC1* expression in a dose-dependent manner. These results suggest that the wild-type GPR peptide induces airway inflammation by stabilizing the Gα_i_-GDP complex, thereby upregulating the production of inflammatory cytokines; the wild-type GPR peptide does not exert the same effects on either MUC1 or TGFβ. However, GPR Q34A peptide modulates airway inflammation in a different manner. We next performed the same experiments using primary cells, specifically, normal human bronchial epithelial (NHBE) cells. These experiments yielded similar results to those obtained with NCI-H292 cells (Fig. 4e).

### The wild-type GPR peptide stimulates CXCR4-induced polymerization of F-actin, whereas the Q34A peptide inhibits cell spreading

**Figure 5:**
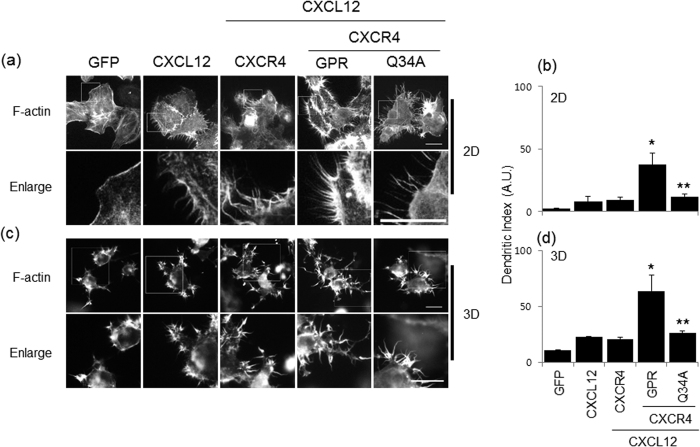
Effect of the GPR peptide on CXCR4-induced F-actin polymerization. Cells were seeded on coverslips and then treated with either the consensus GPR peptide or the Q34A peptide (both at 1.0 μg/ml) prior to treatment with CXCL12 (20 ng/ml). After fixation, rhodamine-conjugated phalloidin was added for 30 min (1:100 dilution). Cells were then stained with DAPI for 2 min (1:10,000 dilution) (**a**). After trypsinizing, the cells are added collagen (1.5 mg/ml; 2 × 10^4^/matrix), and consequently, added media containing FBS and SDF1α was incubated for 24 hours. After blocking with 2% BSA, the cells were incubated with Rhodamine-conjugated phallodin (**c**). The scale bar is 20 μm. Data shown are representative of three independent experiments. (**b,d**) The dendritic index (calculated as perimeter^2^/4π × area) was used to assess morphometric differences between cells. The dendritic index for a round cell was 1.0. *p < 0.05 compared with the control (GFP); **p < 0.05 compared with the treatment of CXCL12 and wild-type GPR peptide.

Dendritic extensions arise through actin polymerization or extension and retraction of lamellipodia^27^. Since CXCR4 signaling is related to chemokine-induced F-actin polymerization^28^, we investigated the effects of the two GPR peptides on CXCR4-induced F-actin polymerization in NCI-H292 cells after treatment with CXCL12. On either 2D (Fig. 5a) or 3D (Fig. 5c) collagen-coated coverslips, the formation of F-actin was increased by formation of the CXCL12/CXCR4 complex. While the consensus GPR peptide dramatically increased the number of dendritic extensions, the Q34A peptide decreased their number. These results suggest that CXCR4 induces CXCL12-mediated F-actin polymerization, consistent with the observation that CXCR4 mediates chemokine-induced movement to control inflammation at the inflamed site. However, the Q34A peptide inhibited dendritic extension to regulate CXCR4 signaling, which may be mediated by the increased MUC1 expression induced by the Q34A peptide. In this context, increased MUC1 production has an anti-inflammatory role in the inflamed microenvironment.

### The GPR Q34A peptide inhibits CXCL12-induced mucous metaplasia, reduces inflammatory cell populations, and decreases inflammatory cytokine production in the mouse lung

**Figure 6:**
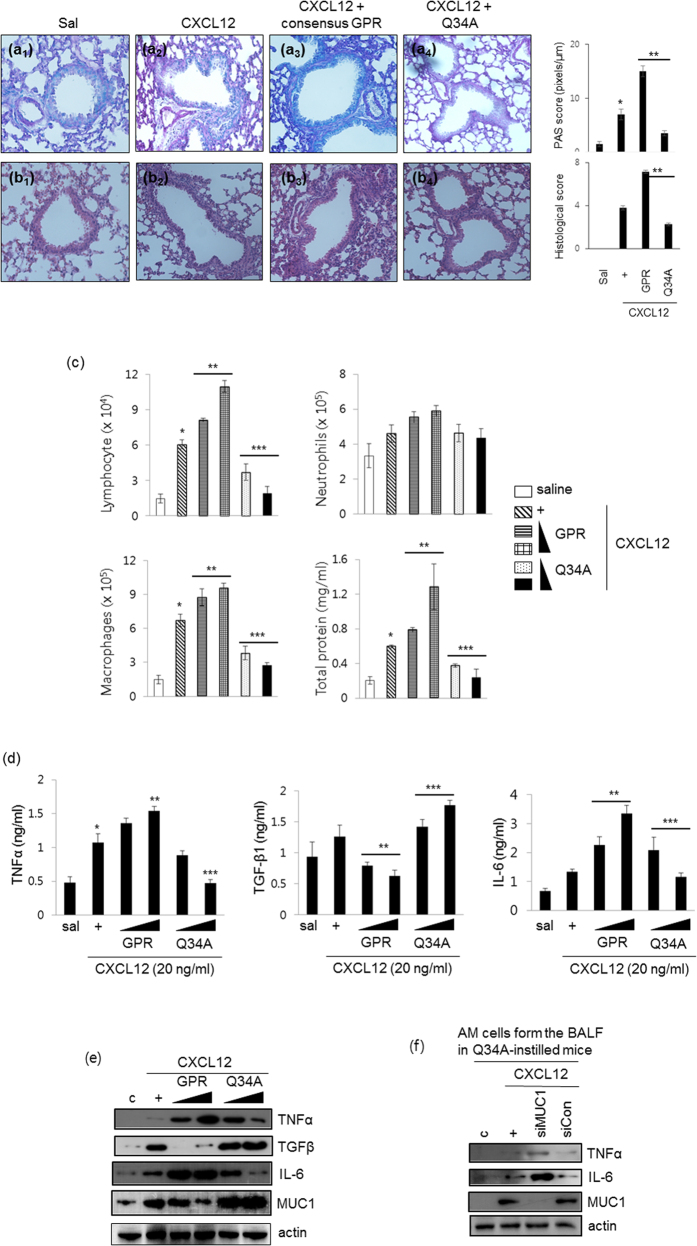
The GPR Q34A peptide suppresses CXCL12-induced lung inflammatory responses *in vivo*. Five days after CXCL12 instillation [30 μl of 50 ng (2.5 μg/kg)] into the tracheal lumens of mice that had been injected with either the consensus GPR peptide or the Q34A peptide (5.0 mg/kg) 24 hours previously, Alcian Blue periodic acid-schiff (AB-PAS) staining (**a**) or hematoxylin and eosin (H&E) staining (**b**) was performed on mouse lung tissue samples (n = 4). *p < 0.05 compared with saline-treated mice; **p < 0.05 compared with CXCL12-treated mice. (**c**) Mice were injected with either the consensus GPR peptide or the Q34A peptide (1.0, 2.5, or 5.0 mg/kg) for 24 hours and then instilled with CXCL12. Mice were sacrificed at five days post-CXCL12 instillation. The levels of lymphocytes, neutrophils, alveolar macrophages, and total protein in the BAL fluid were then determined. *p < 0.05 compared with saline-treated mice; **p < 0.05 compared with CXCL12-treated mice; ***p < 0.05 compared with CXCL12- and consensus GPR peptide-treated mice. (**d**) The levels of TNFα, TGF-β1, and IL-6 in the BAL fluid were measured using ELISA. *p < 0.05 compared with saline-treated mice; **p < 0.05 compared with CXCL12-treated mice; ***p < 0.05 compared with CXCL12- and consensus GPR peptide-treated mice. (**e**) Lungs were harvested from mice treated as in Fig. 6a,b. Total lysates were prepared from lung tissue prior to Western blot analysis. Data shown are representative of three independent experiments. **(f)** The AM cells from the BAL fluid in Q34A-instilled mice were transiently transfected with either siRNA construct of MUC1 or a siRNA control. Cells were treated with CXCL12, and Western blots were subsequently performed. Representative results for more than three independent experiments are shown for each group.

Next, to examine the morphological effects of CXCL12 exposure on the mouse trachea, we performed PAS (Fig. 6a1–4) and H&E (Fig. 6b1–4) staining of CXCL12-treated mice. Five days after CXCL12 instillation into the tracheal lumens of mice, lung tissue was obtained and stained with H&E and PAS. Exposure of mouse lungs to CXCL12 resulted in increased mucous metaplasia and inflammatory cell infiltration and day 5 (Fig. 6a2,b2). Interestingly, these phenomena were much increased by consensus GPR peptide (Fig. 6a3,b3), however, these effects were inhibited dramatically by Q34A peptide instillation (Fig. 6a4,b4). To examine whether the Q34A peptide alters the cell populations in BAL fluid, we quantified various cell populations after instillation of the two GPR peptides but prior to CXCL12 instillation (Fig. 6c). Mice treated with the wild-type GPR peptide had higher levels of lymphocytes, neutrophils, alveolar macrophages, and total protein in BAL fluid; moreover, these effects were mediated by the wild-type GPR peptide in a dose-dependent manner. On the other hand, inflammatory cell populations in the BAL fluid of mice treated with the Q34A peptide had significantly decreased levels of these markers compared with those in CXCL12-instilled mice. To investigate whether the Q34A peptide modulates the production of inflammatory cytokines after instillation of CXCL12, the levels of inflammatory cytokines and anti-inflammatory cytokines were measured using ELISA (Fig. 6d). The levels of TNFα, TGF-β1, and IL-6 in BAL fluid were analyzed three days after CXCL12 treatment. Treatment with the consensus GPR peptide increased CXCL12-induced TNF-α and IL-6 production, whereas treatment with the Q34A peptide decreased the production of these proteins. Interestingly, the level of TGF-β1 was increased in BAL fluid collected from mice treated with the Q34A peptide. Next, we investigated whether the GPR peptides regulate inflammatory cytokine production and MUC1/Muc1 expression in the mouse lung. As expected, the consensus GPR peptide upregulated TNFα and IL-6 expression in the lung, whereas the Q34A peptide upregulated TGF-β and MUC1 expression (Fig. 6e). Lastly, we used siRNA-MUC1 to verify the anti-inflammatory effect of increases in MUC1 expression related to the mutant GPR peptide on inflammatory responses in an animal model. Interestingly, expressions of the TNFα and IL-6 were increased by siRNA-MUC1, suggesting that MUC1 increased/regulated by Q34A peptide could affect these expressions and consequently control inflamed microenvironments (Fig. 6f). Our results show that the GPR Q34A peptide abolishes mucous metaplasia, reduces inflammatory cell populations, and decreases inflammatory cytokine production in both BAL fluid and the lung after CXCL12 instillation.

## Discussion

We performed a detailed investigation of the role of MUC1 during airway inflammation, with special emphasis on the mechanism by which MUC1 modulates inflammation signaling pathways and the role of the GPR motif of AGS3. MUC1/Muc1 was expressed only at low levels in uninflamed cells/lungs; however, MUC1/Muc1 expression was upregulated by CXCL12-induced CXCR4 activation. This finding suggests that CXCR4 is essential for CXCL12-mediated induction of MUC1 expression (Fig. 1). Interestingly, CXCR4 bound MUC1 directly (Fig. 3a), suggesting that the CXCR4/MUC1 interaction is critical in regulating chemokine- and cytokine-mediated induction of MUC1. In the early stages of inflammation, TNFα production is not regulated by MUC1. However, the increased level of MUC1 regulates TNFα production in the late inflammatory response by acting as a regulatory binding protein. Protein-protein interactions are likely an important facet in the regulation of the inflammatory microenvironment during bacterial infection or cytokine-induced airway inflammation. This is the first demonstration that the interaction between CXCR4 and MUC1 plays a role in regulating airway inflammation. MUC1 has previously been reported to bind to the Erb2 receptor in human breast cancer cells^29^; moreover, Muc1 has been shown to interact with EGFR in the mouse mammary gland^30^. Since MUC1 is a transmembrane protein, its binding partners may include transmembrane proteins and/or receptors. Thus, an increased level of MUC1 or of MUC1-receptor/binding partner protein complex may facilitate physiological responses by activating downstream signaling pathways.

CXCR4 is known to be critical for cell migration and final laminar distribution^31^. In addition, CXCL12 is expressed in normal intestinal epithelial cells and has also been shown to be upregulated and differentially distributed in inflammatory bowel disease (IBD)^32^. Based on these studies, we investigated the effect of GPR peptides on CXCL12/CXCR4-induced dendritic distribution in NCI-H292 cells. Visualization of F-actin in cells treated with the wild-type GPR peptide or the Q34A peptide revealed striking morphological differences (Fig. 5). The consensus GPR peptide had a marked effect on F-actin polymerization in 2D culture; the Q34A peptide did not have this effect. These differences suggest that the Q34A peptide-induced upregulation of MUC1 abolishes airway inflammation in the lung, chemokine-induced movement is not necessary for controlling inflammation, and the GPR peptide modulates airway inflammation to control the inflamed microenvironment. Based on our *in vitro* results and our 2D and 3D culture experiments, the Q34A peptide can prevent chemokine-induced airway inflammation. This information is relevant to the development of novel therapeutic drugs for treating respiratory diseases.

We also examined whether the Q34A peptide reduces CXCL12-induced airway inflammation *in vivo*. CXCL12-induced cell populations (lymphocytes, neutrophils, and alveolar macrophages) were increased by the consensus GPR peptide, whereas the Q34A peptide decreased these cell populations. These findings suggest that the Q34A peptide functions as a negative regulator *in vivo* to maintain homeostasis by partially disrupting GDI activity. However, the consequences of the wild-type GPR peptide on CXCL12-induced mucous metaplasia and Muc1 overproduction are still unknown. One possibility is that free Gβγ cannot interact with the GDP-bound Gα_i_-GPR complex, thereby decreasing Muc1 production and upregulating IL-6 and TNFα expression. Qin *et al.* reported that protein-protein interactions between free Gβγ and the C-terminal Gβγ-binding domain of the Ca^2+^ channel α1 subunit inhibited Ca^2+^ channel activity, thus providing an explanation for the functional antagonism of Gβγ^33^. We hypothesize that GDP-bound Gαi released by GPR Q34A peptide binds weakly to free Gβγ, thus restoring Gαi activity. As an alternative possibility, some cytokines may be released extracellularly via CXCR4 after treatment with the cytokine CXCL12 (our unpublished data). These released cytokines/chemokines may modulate immunological responses via GPCR-independent mechanisms. Recently, AGS3-Gα_i2_ bound to G protein-coupled Receptor kinase (GRK) 6 to control CXCR2 expression and function^34^. In addition, knockdown of AGS3 decreased CXCL12 and CCL19-induced migration and immune functions^35^. AGS3 overexpression also decreased CXCL12-induced MUC1 and TNFα expressions in our system (Fig. 2). Interestingly, however, consensus GPR of AGS3 increased airway inflammation (Fig. 6). Therefore, we suggest that GPR Q34A peptide modulates GPCR/G-protein signaling to dramatically decrease mucous cell infiltration and goblet cell metaplasia. Moreover, the increase in Muc1 expression induced by the Q34A peptide controls pathogenesis during airway inflammation.

To further confirm the effects of the GPR peptides on Muc1 expression and cytokine production, we performed *in vivo* studies and examined the mouse lung. The results from these experiments were consistent with the *in vitro* results (Fig. 6e), thereby validating the role of GPR Q34A peptide in mucus hyperproduction and airway inflammation. Although the GPR motif is known to modulate microtubule dynamics and spindle function^36^, we first reported that GPR Q34A peptide exerts an anti-inflammatory synergistic effect with MUC1/Muc1. An abnormal level of MUC1 or defective transcriptional/translational regulation of MUC1 may interfere with the development of chronic inflammatory lung diseases^7^. Therefore, the mutant GPR peptide may be an important tool for regulating inflammatory responses in the airway.

TGFβ has the dual roles of inflammatory cytokines and anti-inflammation in the control of immune diseases.

In the presence of IL-6, TGFβ runs the differentiation of T helper 17 (Th17) cells, which can promote further inflammation and augment autoimmune conditions^37^. TGFβ conducts the differentiation of both Th17 and Treg cells in a dose-dependent manner^38^. In asthma, TGFβ is thought to be particularly involved in the development of subepithelial fibrosis, since TGFβ *in vitro* stimulates fibroblast proliferation, promotes matrix protein synthesis and increases the expression of receptors for extracellular matrix components^39^. Aside from its role in inflammation, the anti-inflammatory effects of TGFβ are exerted by inhibiting the proliferation of T and B lymphocytes, thereby inhibiting Th2 cell function and cytokine production^39^. Moreover, TGFβ induces the deactivation of macrophages and inhibits the survival and differentiation of eosinophils^39^. The exact functions of TGFβ will be determined some immunomodulatory molecule(s) that can combine with TGFβ to decide either inflammation or anti-inflammation.

## Conclusion

**Figure 7:**
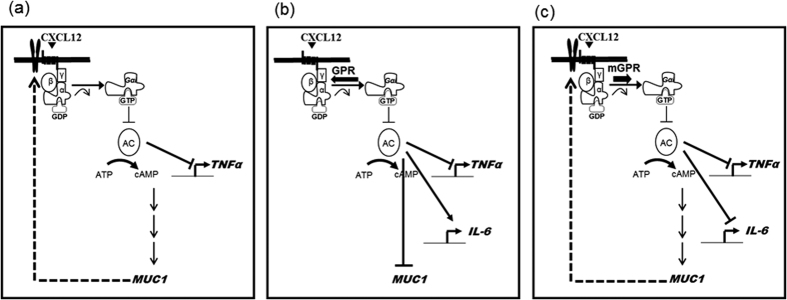
A schematic model illustrating that GPR Q34A peptide inhibits CXCL12/CXCR4-induced *TNFα* and *IL-6* gene expression. The resulting MUC1 interacts with activated CXCR4 to downregulate TNFα expression **(a)**. Wild-type GPR peptide increased IL-6 expression, but not MUC1 and TNFα expression **(b)**. However, the Q34A peptide increases the anti-inflammatory response **(c)**, thereby regulating the inflammatory microenvironment in the airway.”

In conclusion, our study suggests that CXCL12 stimulates MUC1/Muc1 production in airway epithelial cells, mainly through the activation of CXCR4. The resulting MUC1 interacts with activated CXCR4 to downregulate TNFα expression. Overexpressed AGS3 increased IL-6 gene expression, but not MUC1 and TNFα expression. However, the Q34A peptide increases the anti-inflammatory response, thereby regulating the inflammatory microenvironment in the airway (Fig. 7). This is the first report to demonstrate an anti-inflammatory role of the GPR motif of AGS3 and also the first to show a synergistic effect between the GPR motif and MUC1 during airway inflammation. These findings thus provide additional insight into the molecular mechanisms of GPCR/G-protein/G-protein-regulated accessary protein-mediated immune responses in respiratory diseases.

## Materials and Methods

### Materials

CXCL12 (both human and mouse) was purchased from R&D Systems (Minneapolis, MN, USA). Anti-CXCR4 antibodies were purchased from Abcam (Cambridge, MA, USA); anti-MUC1 antibodies were obtained from Pierce (total MUC1) and Abcam (raised against the C-terminal region of MUC1). The following siRNAs were synthesized by Bioneer (Daejeon, Korea): CXCR4, GAUAACUACACCGAGGAAA(dTdT); AGS3, GGGCGCUGGAAUACCACAA(dTdT); and negative control, CCUACGCCACCAAUUUCGU(dTdT). GPR peptides were synthesized by Peptron (Daejeon, Korea).

### Gα_i_ functional assay

cAMP concentrations were measured using a cAMP-Glo assay system (Promega; Fitchburg, WI, USA) according to the manufacturer’s instructions. Briefly, SDF1α-treated cells were added to cAMP-Glo^TM^ lysis buffer, and then incubated at room temperature for 15 min. The cells lysates were added to cAMP-Glo^TM^ reaction buffer, cAMP-Glo^TM^ detection solution, and kinase-Glo reagent, respectively. The level of remaining ATP is determined using the luciferase-based Kinase-Glo^®^ Reagent. Luminescence is inversely proportional to cAMP levels. Thus, as cAMP concentration increases, luminescence decreases.

### Immunoprecipitation

Cells were transfected with either wild-type CXCR4 or siRNA-CXCR4 using FuGENE6. Following transfection, cells were treated with CXCL12 for 4 hours, harvested by scraping into lysis buffer [25 mM HEPES, 150 mM NaCl, 0.5% NP-40, 1 mM EDTA, 1 mM EGTA, and protease inhibitor tablet (Complete Mini; Roche)], sonicated (4 times each for 5 sec), and centrifuged at 12,000 × *g* for 15 min^40^. 230 μL of supernatant lysates were pre-cleared with Dynabeads Protein G (Life Technologies; Oslo, Norway) for 30 min at 4 °C. Following centrifugation, appropriate antisera were added to the pre-cleared lysates, incubated for 30 min at 4 °C, and added Dynabeads Protein G. Immunoprecipitated protein complexes were recovered using DynaMag-2.

### *In vitro* GST pull-down assay

*In vitro* GST pull-down assay was performed using recombinant GST-GPR proteins as exogenous binding partners. Cell lysates prepared from NCI-H292 cells transfected with a construct driving the expression of Gα_i3_ were loaded with 100 μM GTPγS or 100 μM GDP and 25 mM MgCl_2_ at 24 °C for 30 min. The lysates were then incubated with 250 nM GST fusion proteins for 30 min, and the bound proteins were analyzed by immunoblotting with antibodies specific for Gα_i3_ and GST. For adding peptides, Tat-only, Tat-GPR, Tat GPR Q34A, or Tat GPR R30A peptides (100 ng/ml, each) was added for 30 min after treating the cell lyates with 100 μM GDP and 25 mM MgCl_2_ at 24 °C for 30 min. The lysates were then incubated with 250 nM GST fusion proteins for 30 min, and the bound proteins were analyzed by immunoblotting with specific antibodies.

### F-actin staining on 2D collagen-coated coverslips

Cells were seeded on coverslips and transfected with a CXCR4 expression construct prior to treatment with either the consensus or Q34A peptide. Cells were then treated with CXCL12 for 24 hours. After washing, cells were fixed with 3.7% paraformaldehyde for 15 min and then permeabilized for 10 min in 0.5% Triton X-100 solution. After blocking with 2% BSA, cells were incubated with rhodamine-conjugated phalloidin (1:100) for 10 min and then stained with DAPI. For 3D culture on collagen-containing matrices, cells were transfected with the CXCR4 expression construct prior to treatment with either the consensus or Q34A peptide. After trypsinization, cells were added to the collagen matrices (1.5 mg/ml; 2 × 10^4^ cells/matrix). Medium containing FBS and CXCL12 was added to the matrices, and cells were incubated for 24 hours. After blocking with 2% BSA, the cells were incubated with rhodamine-conjugated phallodin. The dendritic index was calculated as perimeter^2^/4π × area^41^,^42^ and used to identify morphometric differences between cells. The dendritic index for a round cell was 1.0.

### Tracheotomy

Six- to eight-week-old C57BL/6 mice were maintained in accordance with the guidelines and under the approval of the Animal Care Committee of Kosin University College of Medicine, Busan, Korea. For intratracheal instillation, mice were anesthetized with Zoletil (30 mg/kg) and Rompun (20 mg/kg) and their tracheas were surgically exposed by making an incision in the neck skin. Peptide (1.0, 2.5, or 5.0 mg/kg/30 μl) was used for injection into the exposed trachea using a microsyringe equipped with a 31-gauge needle. After 24 hrs, CXCL12 solution (50 μg/kg/30 μl) or saline (30 μl) was administrated into the exposed trachea that had been injected with peptide the day before.

### Collection of BAL fluid, measurement of cell populations, and ELISA

BAL was performed by slowly injecting 0.5 ml of ice-cold PBS, through the tracheal tube. The fluid was slowly obtained by gentle suction immediately after delivery, and cell counts were determined using an electronic Coulter Counter fitted with a cell sizing analyzer (Beckman Coulter; Indianapolis, IN). Macrophages and neutrophils cells were identified based on cell diameters. TNFα, TGFβ1, and IL-6 protein levels were measured from the first sample of BAL fluid with specific ELISA kits (R&D Systems).

### Histological analysis

Tissues of mice lung were fixed in 10% formalin and embedded in paraffin. Paraffin-embedded slices were stained with periodic acid-Schiff’s (PAS) solution or hematoxylin/eosin (H&E). Morphometric analysis of PAS-stained sections was performed by quantifying PAS pixels per μm length of bronchial epithelium using the Image J software^43^. At least six areas from similar sections per mouse and at least four mice were estimated randomly. The PAS score for PBS control mouse lung tissue was <2. Histopathologic analysis of inflammatory cells in H&E-stained lung slices from at least four mice was carried out in a blinded manner using a semi-quantitative scoring system^43^.Both peribronchiolar and perivascular inflammation were scored giving a maximum score of 8 as follows^43^: 0, normal; 1, few cells; 2, a ring of inflammatory cells one cell layer deep; 3, a ring of inflammatory cells two to four cells deep; and 4, a ring of inflammatory cells of more than four cells deep. Histological score for PBS control mouse lungs was always 0^43^.

### Statistical analysis

Data are presented as mean ± S.D. of at least three independent experiments. When appropriate, statistical differences were assessed with the Wilcoxon Mann-Whitney test. A *p*-value less than 0.05 was considered statistically significant.

## Additional Information

**How to cite this article**: Choi, I.L.-W. *et al.* Regulation of Airway Inflammation by G-protein Regulatory Motif Peptides of AGS3 protein. *Sci. Rep.* **6**, 27054; doi: 10.1038/srep27054 (2016).

## Supplementary Material

Supplementary Information
