# Supplementary material for: Regulation of Airway Inflammation by G-protein Regulatory Motif Peptides of AGS3 protein
Source: Sci Rep. 2016 Jun 7;6:27054. doi: 10.1038/srep27054 (PMC4895231; doi:10.1038/srep27054)
Supplement: Supplementary Information [file srep27054-s1.pdf]

Supplemental Figures

Regulation of Airway Inflammation by G-protein Regulatory Motif Peptides of AGS3 protein

IL-Whan Choi[1], Do Whan Ahn[2], Jang-Kyu Choi[2], Hee-Jae Cha[3,4], Mee Sun Ock[3], EunAe You[5], SangMyung Rhee[5], Kwang Chul Kim[6], Yung Hyun Choi[7,8], & Kyoung Seob Song[2,4,8]

*Short title: Effect of the GPR motif on airway inflammation

[1]Department of Microbiology, Inje University College of Medicine, Busan, Korea;
[2]Department of Physiology, Kosin University College of Medicine, Busan, Korea;
[3]Department of Parasitology and Genetics, Kosin University College of Medicine, Busan, Korea;
[4]Institute of Medicine, Kosin University College of Medicine, Busan, Korea;
[5]Department of Life Science, Chung-Ang University, Seoul, Korea;
[6]Department of Otolaryngology-Head and Neck Surgery, University of Arizona College of Medicine, Tucson, AZ, USA.
[7]Department of Biochemistry, College of Korean Medicine, Don-Eui University, Busan, Korea;
[8]These authors are contributed equally.

To whom correspondence should be addressed:

Yung Hyun Choi, Department of Biochemistry, College of Oriental Medicine, Don-Eui University, Busan, Korea. Tel: +82-51-850-7413; Fax: +82-51-853-4036; E-mail: choiyh@deu.ac.kr

Kyoung Seob Song, Department of Physiology, Kosin University College of Medicine, 34 Amnam-dong, Seo-gu, Busan 602-703, Korea, Tel.: +82-51-990-6236; Fax.: +82-51-990-3081; E-mail: kssong@kosin.ac.kr

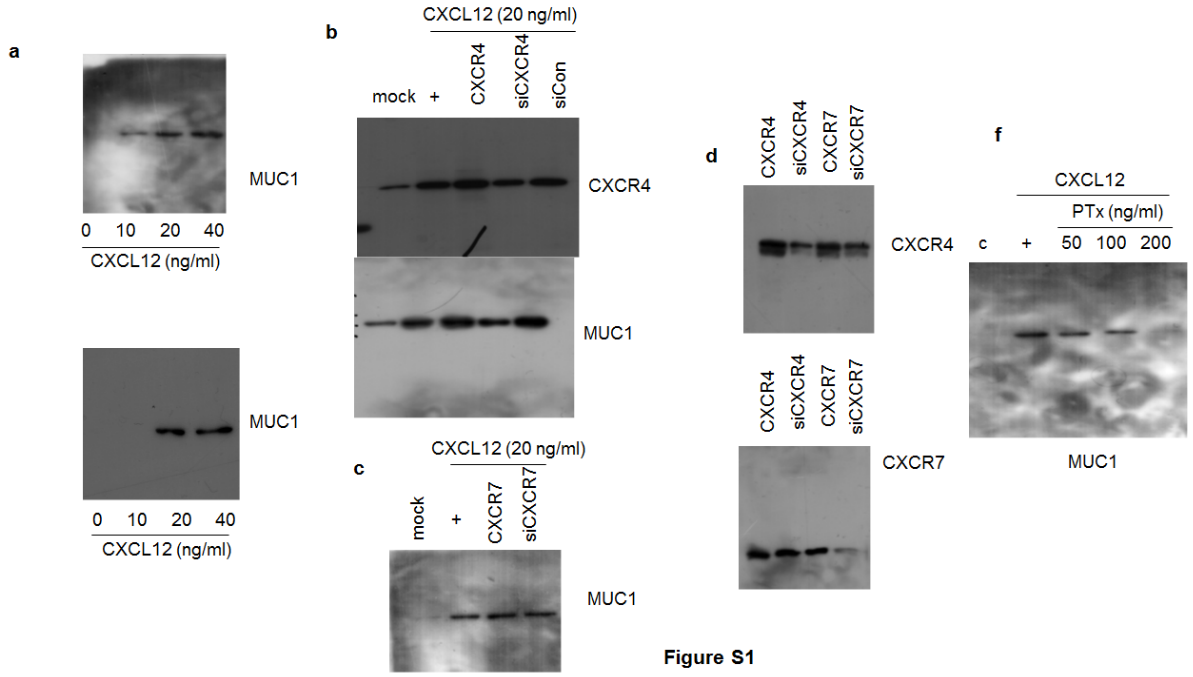
a
MUC1
0 10 20 40
CXCL12 (ng/ml)
MUC1
0 10 20 40
CXCL12 (ng/ml)
b
CXCL12 (20 ng/ml)
mock
+
CXCR4
siCXCR4
siCon
CXCR4
MUC1
c
CXCL12 (20 ng/ml)
mock
+
CXCR7
siCXCR7
MUC1
d
CXCR4
siCXCR4
CXCR7
siCXCR7
CXCR4
CXCR4
siCXCR4
CXCR7
siCXCR7
CXCR7
f
CXCL12
PTx (ng/ml)
c
+
50
100
200
MUC1

Figure S1

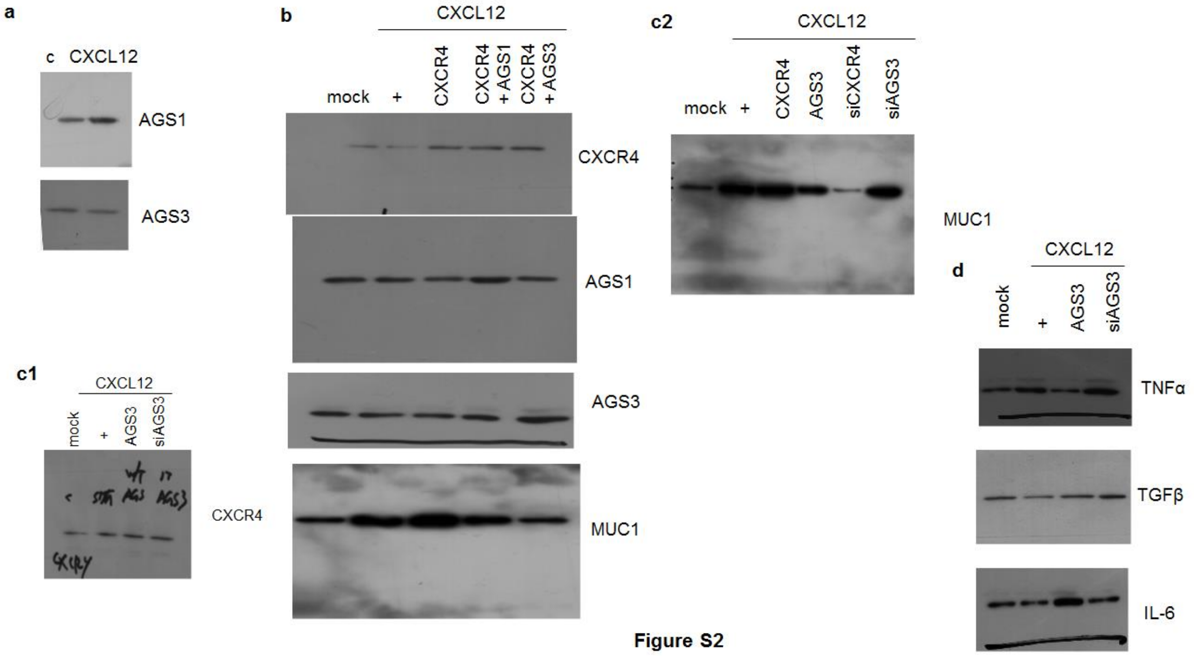
a
c
CXCL12
AGS1
AGS3
b
CXCL12
mock
+
CXCR4
CXCR4 + AGS1
CXCR4 + AGS3
CXCR4
AGS1
AGS3
MUC1
c1
CXCL12
mock
+
AGS3
siAGS3
CXCR4
c2
CXCL12
mock
+
CXCR4
AGS3
siCXCR4
siAGS3
MUC1
d
CXCL12
mock
+
AGS3
siAGS3
TNFα
TGFβ
IL-6

Figure S2

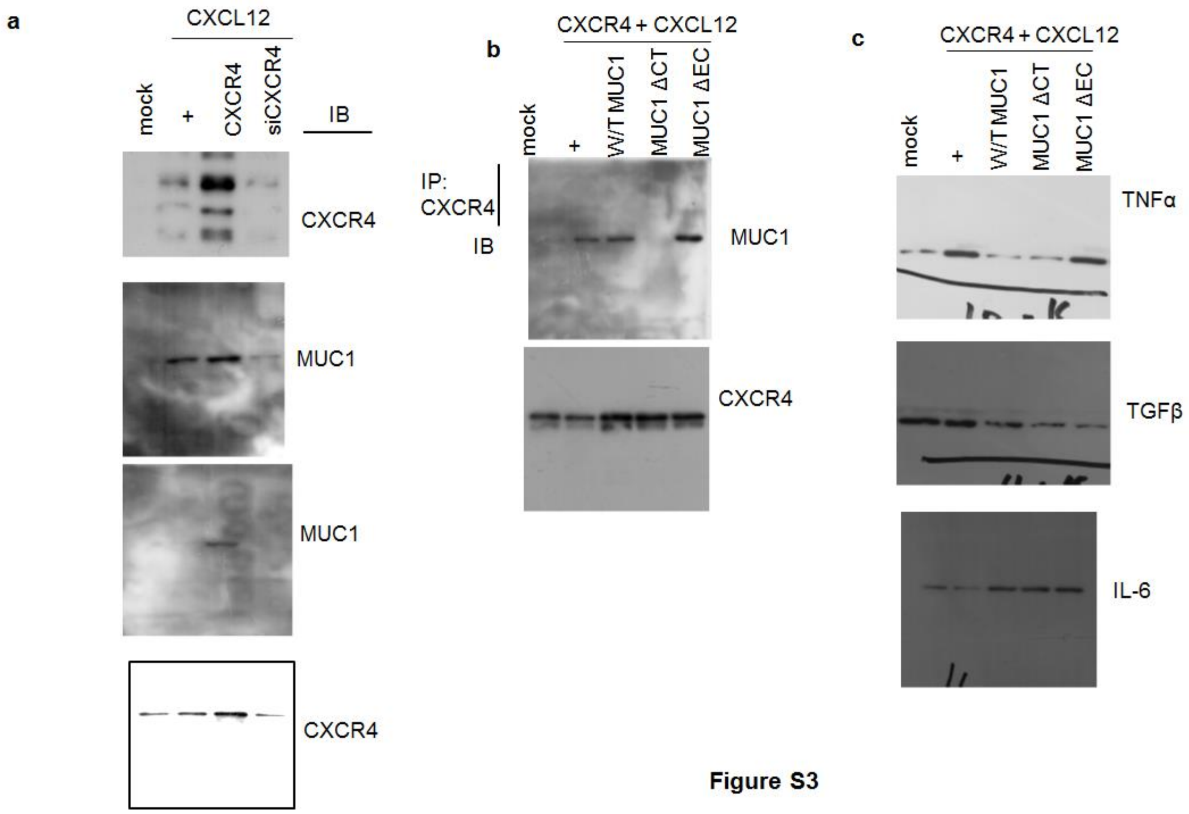
a
CXCL12
mock
+
CXCR4
siCXCR4
IB
CXCR4
MUC1
MUC1
CXCR4
b
CXCR4 + CXCL12
mock
+
W/T MUC1
MUC1 ΔCT
MUC1 ΔEC
IP: CXCR4
IB
MUC1
CXCR4
c
CXCR4 + CXCL12
mock
+
W/T MUC1
MUC1 ΔCT
MUC1 ΔEC
TNFα
TGFβ
IL-6

Figure S3

a

b

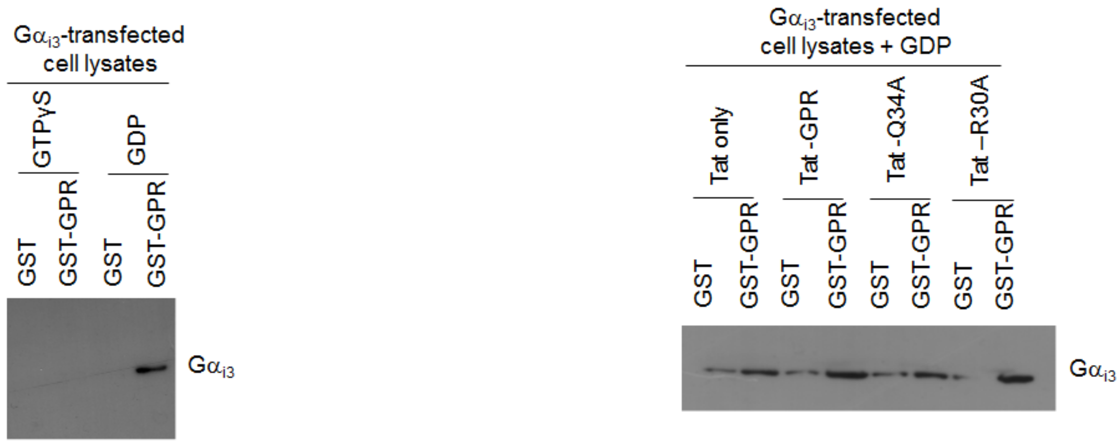
Gαi3-transfected cell lysates
GTPγS
GDP
GST
GST-GPR
GST
GST-GPR
Gαi3
Gαi3-transfected cell lysates + GDP
Tat only
Tat -GPR
Tat -Q34A
Tat –R30A
GST
GST-GPR
GST
GST-GPR
GST
GST-GPR
GST
GST-GPR
Gαi3

Figure S4

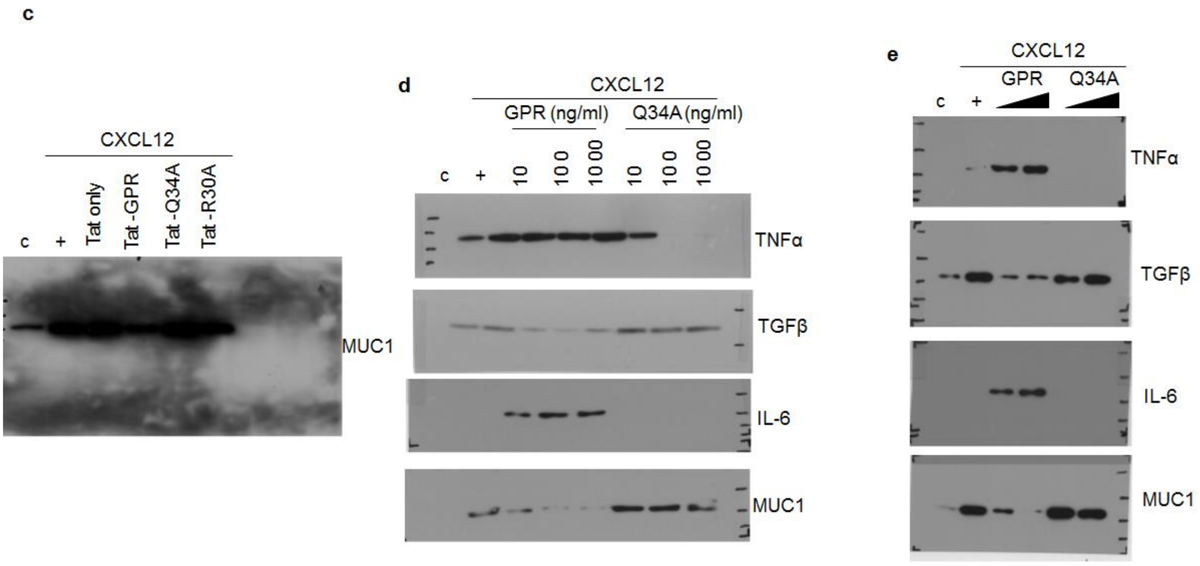
c
CXCL12
c
+
Tat only
Tat -GPR
Tat -Q34A
Tat -R30A
MUC1
d
CXCL12
GPR (ng/ml)
Q34A (ng/ml)
c
+
10
100
1000
10
100
1000
TNFα
TGFβ
IL-6
MUC1
e
CXCL12
GPR
Q34A
c
+
TNFα
TGFβ
IL-6
MUC1

Figure S4

e

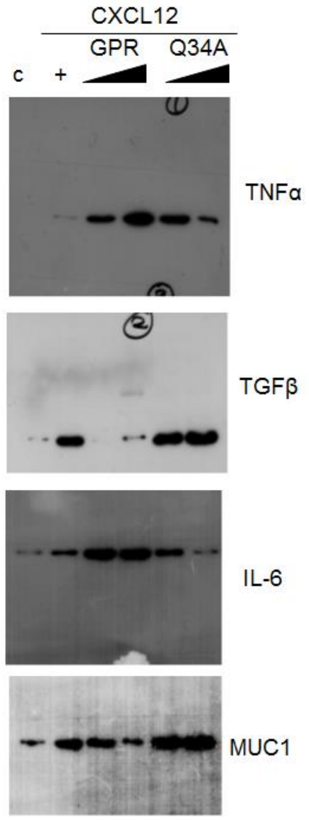

CXCL12
GPR
Q34A
c
+
TNFα
TGFβ
IL-6
MUC1

f

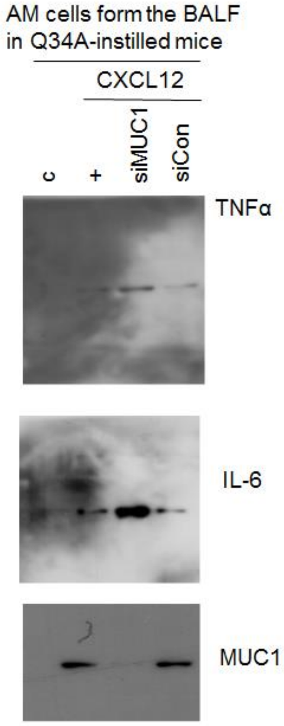

AM cells form the BALF in Q34A-instilled mice
CXCL12
c
+
siMUC1
siCon
TNFα
IL-6
MUC1

Figure S6
